# Correction: Suppressing nonradiative decay *via* molecular configuration control in Cu(i)–halide clusters enables the fabrication of highly efficient green and green-sensitized blue OLEDs

**DOI:** 10.1039/d6sc90060g

**Published:** 2026-03-09

**Authors:** Xiao Li, Sai Guo, Xin Liu, Yu-Fu Sun, Dong-Hai Zhang, Hui Yang, Jia-Min Lu, Xu-Lin Chen

**Affiliations:** a State Key Laboratory of Structural Chemistry, Fujian Institute of Research on the Structure of Matter, Chinese Academy of Sciences Fuzhou Fujian 350002 China xlchem@fjirsm.ac.cn; b Clinical Research Institute, The First Affiliated Hospital of Xiamen University, School of Medicine Xiamen University Xiamen Fujian 361003 China lujiamin@xmu.edu.cn; c Xiamen Key Laboratory of Rare Earth Photoelectric Functional Materials, Xiamen Institute of Rare Earth Materials, Chinese Academy of Sciences Xiamen Fujian 361021 China

## Abstract

Correction for ‘Suppressing nonradiative decay *via* molecular configuration control in Cu(i)–halide clusters enables the fabrication of highly efficient green and green-sensitized blue OLEDs’ by Xiao Li *et al.*, *Chem. Sci.*, 2026, https://doi.org/10.1039/d5sc09307d.

The authors regret that the noted affiliations of the authors were not detailed correctly in the published manuscript. The correct affiliations are shown in the author/affiliation lists here.

The Royal Society of Chemistry apologises for these errors and any consequent inconvenience to authors and readers.

